# A novel long noncoding RNA PILRLS promote proliferation through TCL1A by activing MDM2 in Retroperitoneal liposarcoma

**DOI:** 10.18632/oncotarget.14814

**Published:** 2017-01-25

**Authors:** Yebo Shao, Yong Zhang, Yingyong Hou, Hanxing Tong, Rongyuan Zhuang, Zhengbiao Ji, Binliang Wang, Yuhong Zhou, Weiqi Lu

**Affiliations:** ^1^ Department of General Surgery, Zhongshan Hospital, Fudan University, Shanghai 200032, China; ^2^ Department of Medical Pathology, Zhongshan Hospital, Fudan University, Shanghai 200032, China; ^3^ Department of Medical Oncology, Zhongshan Hospital, Fudan University, Shanghai 200032, China; ^4^ Department of Ultrasound, Zhongshan Hospital, Fudan University, Shanghai 200032, China; ^5^ Department of Radiation Oncology, Zhongshan Hospital, Fudan University, Shanghai 200032, China

**Keywords:** LncRNA-PILRLS, proliferation, MDM2, P53 signaling pathway, retroperitoneal liposarcoma

## Abstract

It is becoming evident that lncRNAs may be an important class of pervasive genes involved in carcinogenesis and metastasis. However, the biological and molecular mechanisms of lncRNAs in retroperitoneal liposarcoma have never been reported. In our study, we found a novel lncRNA PILRLS (Proliferation Interacting LncRNA in Retroperitoneal Liposarcoma), which as an oncogene significantly overexpressed in retroperitoneal liposarcoma. Functions of PILRLS on tumor progression both *in vitro* and *in vivo* have verified in this study which PILRLS knockdown significantly inhibited cell proliferation and colony formation. RNA pull-down assay found PILRLS can specific binding with TCL1A which also regulate the expression level of TCL1A. Our work for the first time demonstrated PILRLS can activating the MDM2 by binding with TCL1A which suppress the P53 pathway to promote the unlimited growth of retroperitoneal Liposarcoma cells. It suggests that PILRLS may be an important targets for retroperitoneal liposarcoma therapy.

## INTRODUCTION

Lipomas are the most common tumor which accounted 15% soft tissue neoplasms by surgical pathologists [1, 2]. Due to its gigantic size and deep location, treatment of retroperitoneal liposarcoma (RLS) is faced with considerable challenges at present. Separating lipomas from well differentiated liposarcomas is usually not difficult in routine practice based on their microscopic appearances. Now, surgical resection is the mainstay of therapy for retroperitoneal sarcoma. But, the majority of patients with high-grade RLS will develop locally recurrent following surgery, and this constitutes the cause of death in most patients [3, 4]. In spite of the progress in chemotherapy, radiotherapy and surgical techniques for RLS in recent years, the survival rate of RLS patients remains unsatisfactory [5, 6]. As a consequence, to explore the new insights and therapy targets based on histology, molecular biology, and systemic treatment were urgently needed in RLS.

Long non-coding RNAs (lncRNAs), a subgroup of non-coding RNAs (ncRNAs), are longer than 200 nucleotides in length and with little protein-coding potential [7]. According to their relative location to genomic elements, lncRNAs are categorized as intergenic, overlapping, intronic, and exonic [8]. The overlapping lncRNAs are subdivided into sense, antisense, and bidirectional according to their transcriptional loci relative to the overlapped transcripts [9]. LncRNAs participate in modulating biological processes through regulating gene expression at almost all levels, including chromatin remodeling, transcription, and post-transcription [9–11]. They have the potential to serve as prognostic indicators and therapeutic targets. Although over 95000 human lncRNAs have been annotated, only a few of them have been functionally characterized. Yet, there have been no systematic profiling studies of lncRNAs in retroperitoneal liposarcoma up until now.

In the present study, through transcriptome microarray analysis, we found a number of lncRNAs dysregulated in RLS compared with paired non-tumor tissues. Among the downregulated lncRNAs, we characterized a novel lncRNA PILRLS (**P**roliferation **I**nteracting **L**ncRNA in **R**etroperitoneal **L**ipo**s**arcoma) in RLS progression. PILRLS interacted with T-cell leukemia 1A protein (TCL1A) in RLS cells, further to develop the proliferative functions through activating AKT serine/threonine kinase 1 (AKT) and MDM2. We demonstrated the PILRLS, which stabilized the TCL1A, suppressing P53 signaling pathway to promoted cell proliferation in RLS.

## RESULTS

### PILRLS, a novel lncRNA, has overexpressed in retroperitoneal liposarcoma

**Figure 1:**
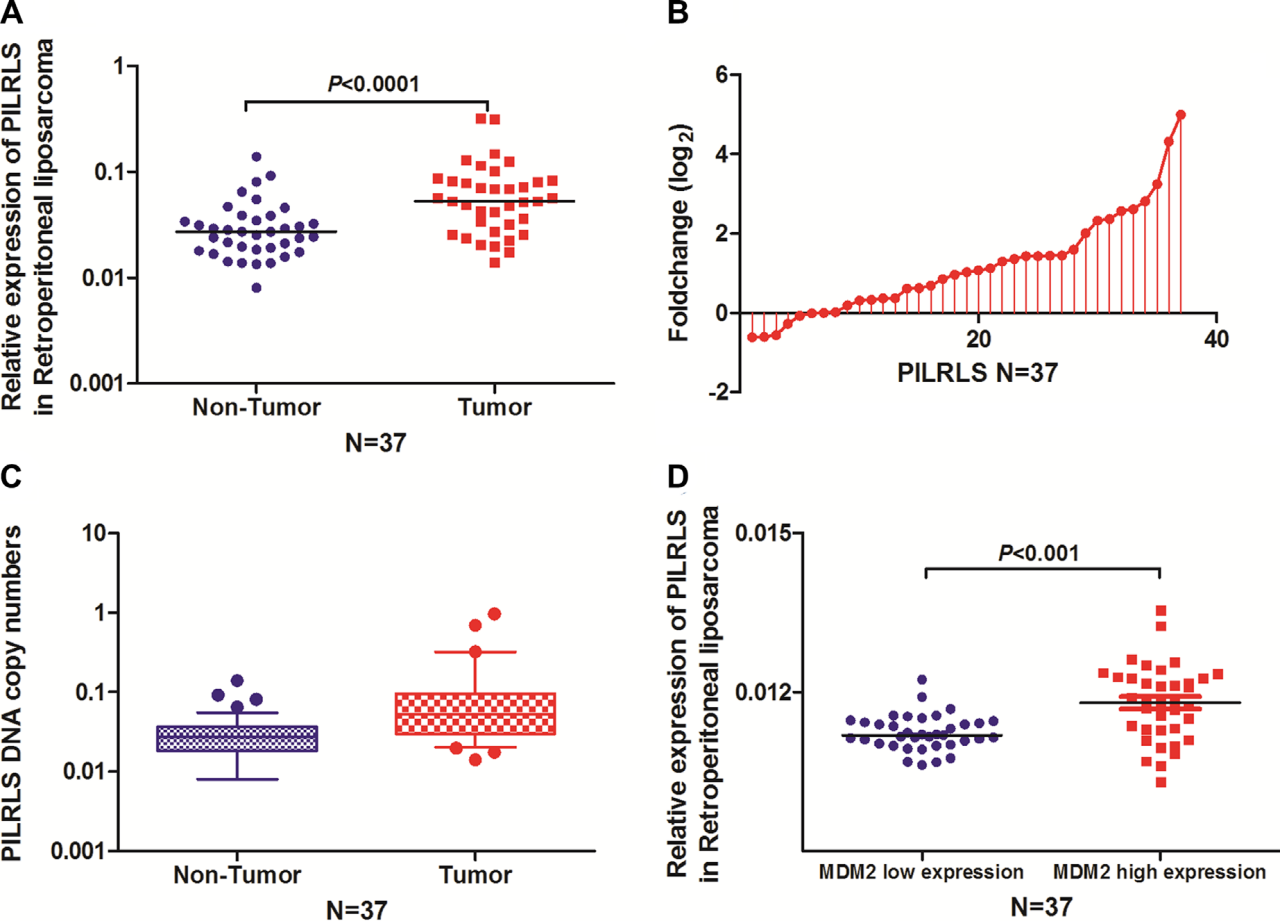
PILRLS overexpressed in RLS as a result of DNA copy number variation (A) Comparison of PILRLS expression levels in 37 matched pairs of RLS tissues and non-tumor tissues (NTs). The qRT-PCR results showed that PILRLS expression was significantly upregulated in tumor tissues. (B) Fold change of PILRLS in 37 paired of RLS tissues and non-tumor tissues (NTs). (C) PILRLS copy numbers were determined for 37 matched RLS tissues and non-tumor tissues (NTs) using qPCR. The copy number of PILRLS was increased in the tumor tissues compared with the NCTs. (D) PILRLS expression was positively correlated with the MDM2 in 37 paired RLS tissue and the NT tissues.

To search for potential carcinogenic lncRNAs involved in retroperitoneal liposarcoma, we globally analysed the lncRNA expression profiles of normal enterocoelia tissues, and retroperitoneal liposarcoma tissues. The expression pattern of the novel lncRNA CTD-2187J20.1 was validated using quantitative reverse transcription PCR (qRT-PCR) analysis in 37 paired of tissues. We found it significantly overexpressed in RLS tissues (Figure 1A and 1B) with gain of copy numbers in RLS (Figure 1C) and positively related with MDM2 level (Figure 1D) which indicate it has the closely relationship with proliferation in RLS. Search the CTD-2187J20.1 in National Center for Biotechnology Information (NCBI) RefSeq, we found it located at chr5:66,563,850–66,566,010 with full-length of 509bp (Supplementary Figure 1A). In consideration of this lncRNA related with proliferation in RLS, we focused our research on CTD-2187J20.1 and renamed it as PILRLS (Proliferation interacting LncRNA in RLS). Using the CPC (Coding Potenital Calculator: http://cpc.cbi.pku.edu.cn/ ) for annotating human lncRNA genes, we classified PILRLS as an lncRNA, as the transcript had no protein-coding potential (Supplementary Figure 1B). Next, we performed a rapid amplification of cDNA ends analysis to identify the 5′ and 3′ ends of the PILRLS transcript. The transcription start and termination sites and Northern-blot analysis the sequences of full-length PILRLS cDNA are presented in (Supplementary Figure 1C and 1D). We have separated the nuclear and cytoplasm fractions of 94T778 RLS cells and performed real-time PCR. We found PILRLS was located both in nucleus and cytoplasm (Supplementary Figure 1E). We also analysis the expression level of PILRLS in three kinds of RLS cell lines (Supplementary Figure 1F), and selected 93T449 and 94T778 for further study. In brief, PILRLS is a novel lncRNA and highly expressed in RLS tissues.

### PILRLS is an oncogenic lncRNA in retroperitoneal liposarcoma *in vitro* and *in vivo*

**Figure 2:**
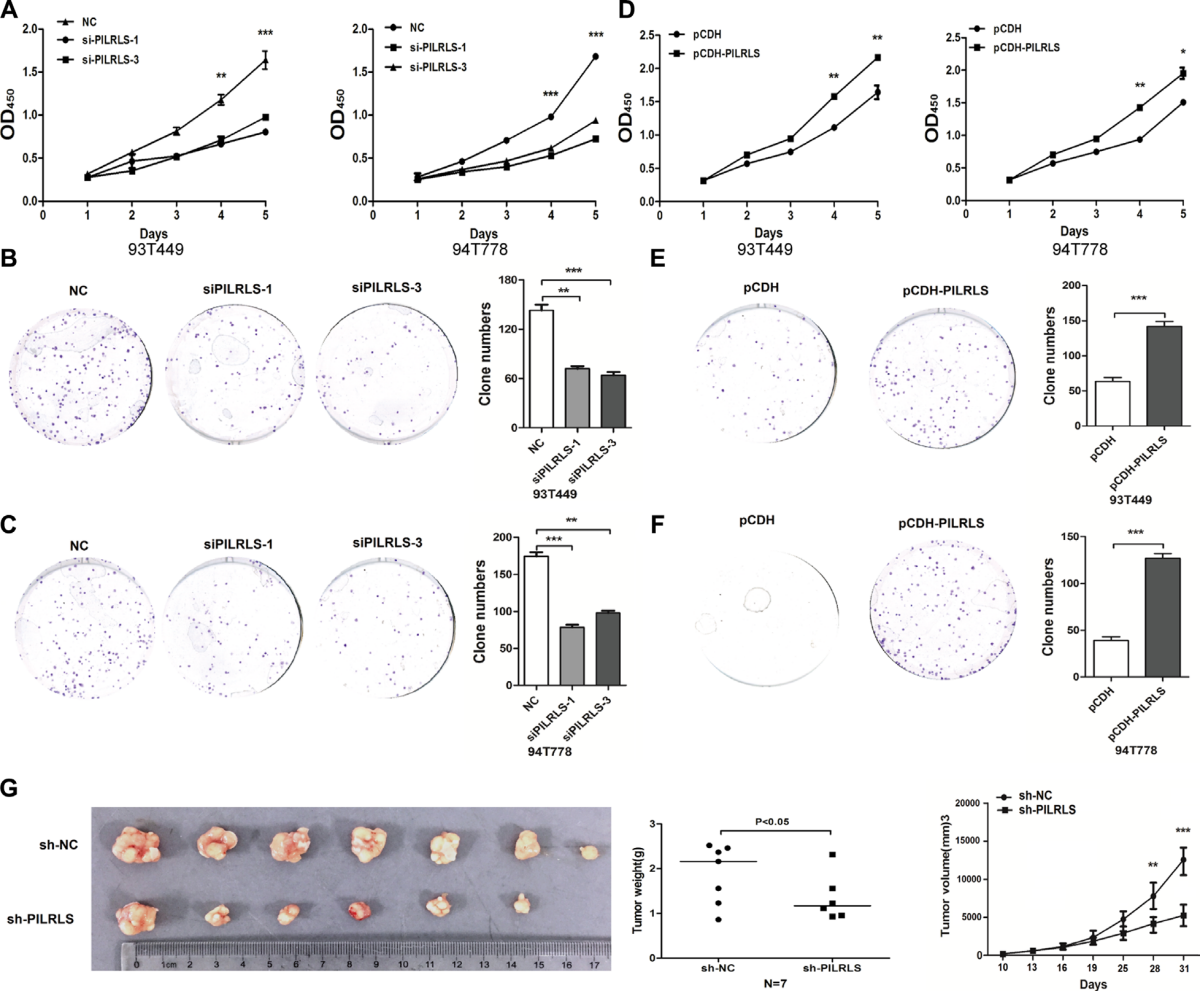
PILRLS promote RLS cell proliferation and colony formation in vitro and in vivo (A) CCK-8 assay to detected knockdown of PILRLS decrease the proliferation ability in 93T449 and 94T778 cancer cells (***P < 0.001). (B–C) Knockdown of PILRLS decreased colony formation in 93T449 and 94T778 cancer cells. The colonies was evaluated by crystal violet staining and counted (***P < 0.001). (D) CCK-8 assay to detected overexpression of PILRLS increase the proliferation ability in 93T449 and 94T778 cells (***P < 0.001). (E–F) Overexpression of PILRLS increased colony formation in 93T449 and 94T778 cells. The colonies was evaluated by crystal violet staining and counted (***P < 0.001). (G) The effect of PILRLS on proliferation in a nude mouse xenograft model. Lentiviral vector-NC and si-PILRLS infected 94T778 cells (1 × 107) were injected into the nude mouse. The tumor size, tumor weight and tumor volume of the si-DMBT1 group was significantly decreased compared with the control group (*** P < 0.001).

To evaluate the oncogenic properties and effects of PILRLS in RLS, we established PILRLS knockdown or stable overexpression in 93T449 and 94T778 RLS cell lines. Knockdown PILRLS expression both in 93T449 and 94T778 cells leads to a significant decrease in cell proliferation and colony formation (Figure 2A–2C). On the other hand, PILRLS stable overexpressed in pCDH-PILRLS 93T449 and 94T778 cells leads to a significant increase in proliferation and colony formation (Figure 2D–2F). To determine the effects of PILRLS on tumorigenesis *in vivo*, PILRLS -shRNA 93T449 cells and appropriate negative control cells were subcutaneously injected into nude mice. Knockdown PILRLS significantly decreased tumor growth, tumor weight and tumor volume *in vivo* compared with the control group. These data suggest that PILRLS as an oncogenic lncRNA in RLS cells and plays an important role in RLS progression.

### PILRLS can specific binding with TCL1A in RLS

**Figure 3:**
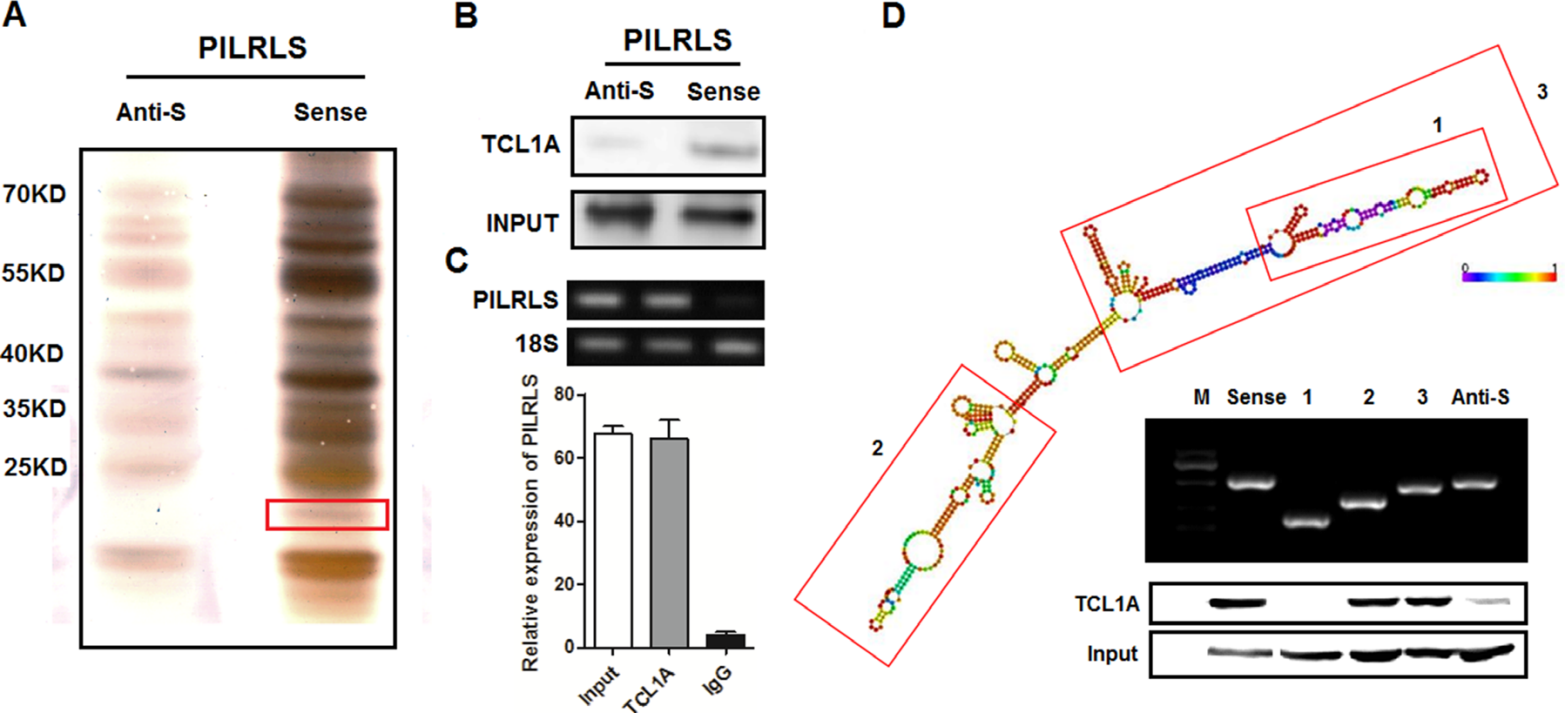
PILRLS specific interact with TCL1A (A) RNA pull-down was performed using a full-length of PILRLS and anti-sense of PILRLS and RNA-binding protein separated by SDS-PAGE in 94T778 cells. The protein bands were excised and detected by mass spectrometry analysis. (B) Western-blot to analysis the interaction of PILRLS and TCL1A using RNA pull-down cell extract. (C) RIP analyses were performed using antibodies against TCL1A, with IgG as a negative control in 94T778 cells. The enrichment of the PILRLS was detected using RT-PCR and normalized to the input. (D) Deletion mapping of the PILRLS according to the second structure. TCL1A was detected by the Western blotting assay in the samples pulled down by fragment of PILRLS.

Previous studies have demonstrated that many lncRNAs regulate molecular pathways via their interactions with proteins [12–15]. To test whether PILRLS affect the biological behaviours of RLS cells in a similar way, we using an RNA-pull-down assay to identify the proteins that are interacting with PILRLS (Figure 3A). Among all of the proteins identified by mass spectrometry, only TCL1A (Approximately 15KD size) was detected by western blotting from three independent RNA pull-down assays (Figure 3B). We further performed RNA immunoprecipitation (RIP) with an antibody against TCL1A using cell extracts from the 94T778 cells. We observed more PILRLS enrichment using the TCL1A antibody than a non-specific antibody (IgG control) (Figure 3C). Next, we explored the molecular consequences of PILRLS and TCL1A association. We further tested the deletion-mapping assays of PILRLS, in LNCipedia database (http://www.lncipedia.org/) showed the second structure of PILRLS (Figure 3D). According to the structure of PILRLS, we constructed the three biotinylated fragments of PILRLS (1, 2, 3) for RNA pull-down assay. We found the 3′ fragment of PILRLS mediated the interaction with TCL1A in RLS cells. Taken together, these results demonstrated a specific association between RILRLS and TCL1A.

### PILRLS suppress the P53 pathway by activing TCL1A and MDM2

**Figure 4:**
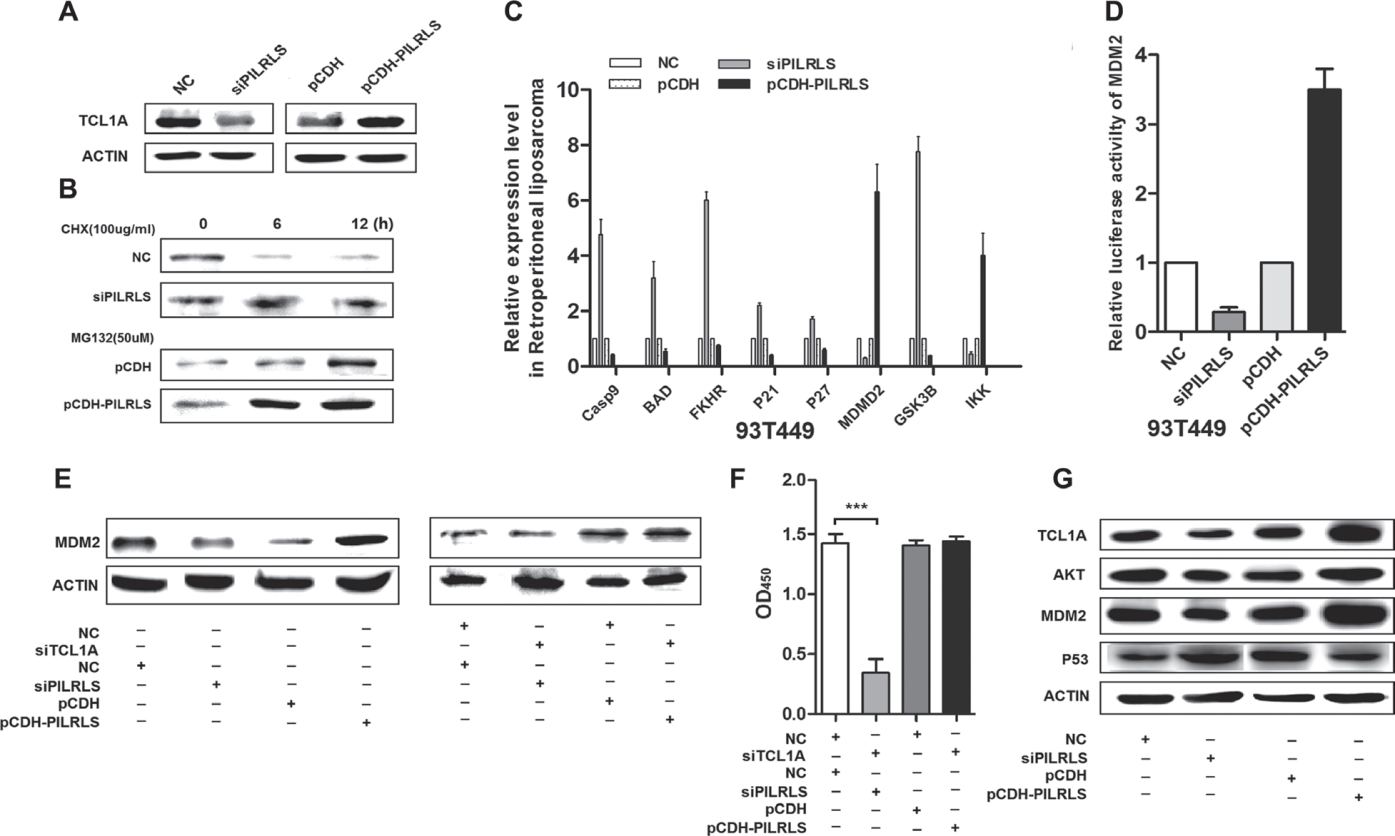
The molecular mechanism of PILRLS promote proliferation in RLS cells (A) Western-blot to detect the protein level of TCL1A whether regulated by PILRLS. (B) 94T778 cells with stable overexpression of PILRLS or knockdown of PILRLS were treated with protein synthesis inhibitor cycloheximide (CHX, 100 ug/ul) or the proteasome inhibitor MG-132 (50uM) for 24 h. Detect the protein level by western-blot. (C) RT-qPCR was used to detect the mRNA levels of Casp9, BAD, FKHR, P21, P27, MDM2, GSK3B and IKK in knockdown and overexpression PILRLS cells. (D) Luciferase activity of MDM2 in knockdown and overexpression PILRLS cells. (E) Western-blot detect whether the protein level of MDM2 was regulated by TCL1A and PILRLS. (F) CCK-8 assay for verify the function of TCL1A and PILRLS. (G) Western-blot to detect the protein level of TCL1A, AKT, MDM2 and P53 when knockdown and overexpression PILRLS.

We have demonstrated PILRLS has the positively relationship with MDM2 in 37 paired tissues. Aberrant P53 signaling pathway plays an important role in proliferation. To detected whether PILRLS-mediated regulation of TCL1A in P53 pathway, we analysis protein level of TCL1A when knockdown and overexpression PILRLS. Results showed (Figure 4A), knockdown of PILRLS can significantly reduce the protein level of TCL1A; on other hand, overexpression of PILRLS, increase the protein level of TCL1A. To determine whether PILRLS increased TCL1A protein stability, we treated si-PILRLS, pCDH-PILRLS and control cells with the protein synthesis inhibitor cycloheximide (CHX, 100ug/ul) or the proteasome inhibitor MG-132 (50uM). As shown (Figure 4B), high level of PILRLS can increased the stability of TCL1A in RLS cells. In addition, we found that overexpression of PILRLS upregulated MDM2 and IKK which, in turn, knockdown of PILRLS reduce the expression of MDM2 and IKK (Figure 4C). Meanwhile, knockdown of PILRLS upregulated Casp9, BAD, FKHR, P21, P27 and GSK3B mRNA level, decreased these genes expression when overexpressed PILRLS (Figure 4C). To understand the regulation mechanism of PILRLS with MDM2, we performed the luciferase reporter arrays. We found that when overexpression of PILRLS, significantly strengthen the relative luciferase activity of MDM2 (Figure 4D). These results illustrated PILRLS can activating the MDM2 through stabilization of TCL1A which as an important modulator in P53 pathway. Results in Figure 4E also demonstrated protein level of MDM2 was regulated by PILRLS. Knockdown of PILRLS lead to the decreased of MDM2, and in contrast, overexpressed PILRLS result in increased of MDM2 protein. Fellow on this phenomenon, knockdown of TCL1A in si-PILRLS cells, the expression of MDM2 was significantly reduced compared with si-PILRLS only. And knockdown of TCL1A in stable-overexpression PILRLS cells, lead to a lower expression of MDM2 contrast overexpressed PILRLS only. Fellow that, we also test the proliferation ability when both knockdown of PILRLS and TCL1A and knockdown TCL1A in pCDH-PILRLS cells (Figure 4F), when knockdown of both, the proliferation ability of RLS cells was dramatic decreased; and this phenomenon was restored when overexpression of PILRLS.

Conclusion that both PILRLS and TCL1A were as oncogenes in RLS, they make a meaningful contribution to cell proliferation. MDM2, as an important number of P53 pathway, so we test the other members include AKT and P53 whether regulated by PILRLS. When knockdown of PILRLS, the protein level of TCL1A, AKT and MDM2 were decreased, the expression of P53 was increased, and when overexpressed PILRLS show the opposite results (Figure 4G). We can safely to conclude that, PILRLS specific binding with and stabilized TCL1A which activating MDM2 and AKT, suppressing P53, leading to promote proliferation in RLS.

These results not only confirm the oncogenic activity of PILRLS *in vitro* and *in vivo*, but also suggest that targeting PILRLS may represent an approach in retroperitoneal liposarcoma treatment.

## DISCUSSION

Until now, discovery of non-coding RNAs, made the enormous contribution to cancer research. Furthermore, while over 32.5% of the genome is affected by CNV (copy number variation), with only 2% of the human genome encodes proteins [7]. These findings, strongly illustrate that non-coding RNAs in CNVs play significant roles in tumor development. However, large-scale functional characterization of lncRNA CNVs is still lacking [16]. Besides, most retroperitoneal soft tissue sarcomas (RPS), even of important size, rarely metastasize which only about 10% of RPS are found to have metastatic disease at presentation, which is mostly hematogenous and equally distributed to the lungs or the liver growth without control [17]. The molecular mechanism underlying the deregulation of cancer-associated lncRNA in retroperitoneal liposarcoma were need more research.

In this study, we analysis the novel lncRNA-PILRLS of 37 paired of RLS and normal enterocoelia tissues. PILRLS, displayed a remarkable trend of increased expression levels with the progression from normal enterocoelia tissues to carcinoma. Our findings suggested that PILRLS plays an important role during RLS tumorigenesis. In this regard, our data contribute to a growing body of literature supporting the importance of non-annotated lncRNA species in the field of cancer research.

We investigated the mechanisms by which PILRLS exerts its function and modulates malignantly RLS used loss of function and gain of function assays. Our data clearly indicated that silencing PILRLS expression inhibited RLS cell proliferation, colony formation both *in vitro* and *in vivo*. The PILRLS transcript was found to be associated with TCL1A to promote the stability of TCL1A protein. We also found that the ability of PILRLS to enhance proliferation is in large part attributed to its ability to activate MDM2 and subsequent suppression of the P53 signaling pathway. These findings provide additional evidence that PILRLS plays an important role in RLS tumorigenesis and progression.

Recent studies have demonstrated the functional roles of lncRNAs, and provided insights into the molecular mechanisms by which lncRNAs function in a variety of human tumors [18, 19]. However, the mechanisms regulating lncRNA expression in RLS have not been thoroughly elucidated. Besides, with a key role for the ubiquitin ligase MDM2, which both targets p53 for degradation and directly inhibits p53 activity by binding to the transcriptional activation domain. Under conditions of cellular homeostasis, MDM2 functions in combination with its binding partner MDMX (also known as MDM4) to keep p53 activity under control. Importantly, p53 transcriptionally activates several of its own regulators, including MDM2, allowing for efficient feedback control to limit the p53 response [20]. Our present results indicate that MDM2 can specific bind with PILRLS, which enriched the molecular mechanisms of MDM2-P53 pathway.

To our knowledge, this is the first study reporting the effects of lncRNAs on retroperitoneal liposarcoma research and the TCL1A-PILRLS interaction, which highlights the association of lncRNAs activating MDM2 in cancer and opens up a new field for lncRNA study. Taken together, our results indicate that PILRLS is an oncogenic lncRNA that promotes the tumorigenesis, suggests that lncRNAs may be important targets for retroperitoneal liposarcoma therapy.

## MATERIALS AND METHODS

### Patients and tissue samples

Human tissues samples (37 cases of retroperitoneal liposarcoma patients) were from the tissue bank at the Department of Pathology, from Feb 2014 to Nov 2015, Zhongshan Hospital affiliated with the Fudan University (Shanghai, China). In our study, all patients without chemotherapy or radiotherapy before surgery. Our study was approved by the Institutional Review Board of Fudan University, and all of the participants signed an informed consent form. Normal human enterocoelia tissue was obtained from patients undergoing resection for RLS and samples were taken from regions several centimeters away from the tumor. All collected tissue samples were immersed in RNA Later stabilization solution (Qiagen, Hilden, Germany) and were immediately frozen in liquid nitrogen and stored at –80°C until RNA isolation.

### Cell culture

HEK293T, 93T449 and 94T778 cells were cultured in complete DMEM medium (LifeTechnologies, Carlsbad, CA, USA) supplemented with 10% fetal bovine serum and penicillin-streptoMycin (Gibco). All cells were cultured at 37 °C with 5% CO2.

### siRNA or overexpressed PILRLS plasmids construct and Quantitative real-time PCR

The small interfering RNA (siRNA) sequences targeting PILRLS and negative control were synthesized by Invitrogen (Shanghai, China). The PILRLS sequence was synthesized according to the sequence in UCSC (University of California Santa Cruz) database. Both were subcloned into a pCDH vector (Invitrogen, Shanghai, China). Cells were transfected at a final siRNA concentration of 50 nM using Lipofectamine 2000 (Life Technologies, Carlsbad, CA, USA) according to the manufacturer's protocol. Cells were collected 24 h or 48 h after the transfection for RNA isolation and 72 h after transfection for western blot analysis. Total RNA was extracted from cells following the manufacturer's instructions with TRIzol (Life Technologies, Carlsbad, CA, USA). Reverse transcription was carried out with PrimeScript™ Reverse Transcriptase Kit (Takara, Dalian, China) and QRT-PCR was performed using the Fast SYBR Green Master Mix (Takara, Dalian, China) on a 7900 HT Fast Real-Time PCR System (Applied Biosystems, Foster City, CA, USA). All reactions were run in triplicate and the comparative cycle threshold (CT) method was applied to quantify the expression level of PILRLS. Results were normalized to the expression of β-actin. The relative expression was calculated by normalizing to the β-actin expression level as control genes. For PILRLS qRT-PCR, the primer pair 5′-TAACTCAAAGGAGGGGAGAAAAG-3′ (forward) and 5′-GATGAATGGCAGGATGAAGGTC -3′ (reverse) was used to amplify a 193-bp product. Human β-actin was using primers 5′-TTCACCACCATGGAGAAGGC-3′ (forward) and 5′-TGCATGGACTGTGGTCATGA-3′ (reverse) as the loading control. The relative amount of PILRLS was calculated using the equation2-^ΔΔCT^.

### Proliferation and colony formation assay

We use Cell Counting Kit 8 (CCK-8, Dojindo, Kumamoto, Japan) to assess the relative cell viability at 1, 3, 5 days after transfection. For the cells to form colonies, a total of 1500 transfected cells and control cells were placed onto a fresh six-well plate and maintained in media containing 10% FBS, media was replaced every 4 d. After 10–14 days, the colonies were fixed with methanol and stained with 0.1% crystal violet (Sigma, St. Louis, MO). Visible colonies were manually counted. Triplicate wells were assessed for each treatment group.

### Western blot

Cells were harvested in RIPA buffer, and boiled for 10 min at 100°C in SDS-loading buffer. 15μg of protein was loaded on a 10% Bis-Tris PAGE gel (NuPAGE Novex) and transferred onto a PVDF membrane. Membrane was blocked in 5% milk and incubated overnight at 4°C with total TCL1A, AKT, MDM2, P53 and β-actin (Santa Cruz Biotechnology, USA) antibody. Membrane was washed, incubated with secondary HRP conjugated antibody and developed using ECL reagent (Pierce, USA).

### 5′and 3′rapid amplification of cDNA ends (RACE) analysis, Subcellular fractionation analysis and assessment of protein-coding potential

We use SMART™ RACE cDNA Amplification Kit (Takara, Japan) to amplification the 3′ and 5′ end of PILRLS according to the manufacturer's protocol. Subcellular fractionation analysis were performed use Nuclear/Cytosol Fractionation Kit (Pierce, USA). We predicted the protein-coding potential of PILRLS using Coding Potential Calculator (CPC) software.

### Northern blot

We used Northern blotting was performed by using NorthernMax Kit from Ambion (Life Technologies) to detect the full-length of PILRLS according to the manufacturer's protocol. In brief, Digoxin-labelled RNA probes were prepared with DIG Northern starter Kit (Roche, Indianapolis, IN, USA) with the PCR products as templates for T7 transcription. 50 mg total RNA run on a 2% agarose gel and transferred to a Hybond-Nþmembrane (GE Healthcare, Uppsala, Sweden). At last, the blot was visualized by phosphorimaging (Typhoon, Molecular Devices).

### Xenograft transplantation assay

Approximately 5.0*10^6^ 93T449 cells suspended in 100 μl PBS and stably transfected with shRNA-PILRLS or shRNA/control were injected subcutaneously into the right side of the posterior flank of female BALB/c athymic nude mice (Department of Medicine, Fudan University) at five to six weeks of age. Tumor growth was examined every other day with a vernier caliper. Tumor volumes were calculated by using the equation: V = A*B^2^/2 (mm^3^), where A is the largest diameter and B is the perpendicular diameter. After five weeks, all mice were killed and necropsies were carried out.

### Statistical analysis

Statistical analyses were performed using GraphPad Prism 6.0 (Graphpad Software Company, USA). Statistically significant differences were calculated using Student's *t*-test, Wilcoxon rank-sum test, Mann–Whitney *U*-test and Pearson's correlation, as appropriate. *P*-value less than 0.05 were considered as statistically significant.

## SUPPLEMENTARY MATERIALS FIGURES AND TABLES



## References

[1] Taguchi S, Kume H, Fukuhara H, Morikawa T, Kakutani S, Takeshima Y, Miyazaki H, Suzuki M, Fujimura T, Nakagawa T, Ishikawa A, Igawa Y, Homma Y (2016). Symptoms at diagnosis as independent prognostic factors in retroperitoneal liposarcoma. Molecular and clinical oncology.

[2] Torre LA, Bray F, Siegel RL, Ferlay J, Lortet-Tieulent J, Jemal A (2015). Global cancer statistics, 2012. CA.

[3] Zhao X, Li P, Huang X, Chen L, Liu N, She Y (2015). Prognostic factors predicting the postoperative survival period following treatment for primary retroperitoneal liposarcoma. Chinese medical journal.

[4] Tseng WW, Malu S, Zhang M, Chen J, Sim GC, Wei W, Ingram D, Somaiah N, Lev DC, Pollock RE, Lizee G, Radvanyi L, Hwu P (2015). Analysis of the intratumoral adaptive immune response in well differentiated and dedifferentiated retroperitoneal liposarcoma. Sarcoma.

[5] Vijay A, Ram L (2015). Retroperitoneal liposarcoma: a comprehensive review. American journal of clinical oncology.

[6] Matthyssens LE, Creytens D, Ceelen WP (2015). Retroperitoneal liposarcoma: current insights in diagnosis and treatment. Frontiers in surgery.

[7] Li CH, Chen Y (2016). Insight Into the Role of Long Noncoding RNA in Cancer Development and Progression. International review of cell and molecular biology.

[8] Schmitt AM, Chang HY (2016). Long Noncoding RNAs in Cancer Pathways. Cancer cell.

[9] Zhou T, Kim Y, MacLeod AR (2016). Targeting Long Noncoding RNA with Antisense Oligonucleotide Technology as Cancer Therapeutics. Methods in molecular biology.

[10] Wilusz JE (2016). Long noncoding RNAs: Re-writing dogmas of RNA processing and stability. Biochimica et biophysica acta.

[11] Chen LL (2016). Linking Long Noncoding RNA Localization and Function. Trends in biochemical sciences.

[12] Jiang F, Zhou X, Huang J (2016). Long Non-Coding RNA-ROR Mediates the Reprogramming in Cardiac Hypertrophy. PLoS One.

[13] Chang L, Wang G, Jia T, Zhang L, Li Y, Han Y, Zhang K, Lin G, Zhang R, Li J, Wang L (2016). Armored long non-coding RNA MEG3 targeting EGFR based on recombinant MS2 bacteriophage virus-like particles against hepatocellular carcinoma. Oncotarget.

[14] Kotake Y, Kitagawa K, Ohhata T, Sakai S, Uchida C, Niida H, Naemura M, Kitagawa M (2016). Long Non-coding RNA, PANDA, Contributes to the Stabilization of p53 Tumor Suppressor Protein. Anticancer Res.

[15] Yuan JH, Yang F, Wang F, Ma JZ, Guo YJ, Tao QF, Liu F, Pan W, Wang TT, Zhou CC, Wang SB, Wang YZ, Yang Y (2014). A long noncoding RNA activated by TGF-beta promotes the invasion-metastasis cascade in hepatocellular carcinoma. Cancer cell.

[16] Jia D, Wei L, Guo W, Zha R, Bao M, Chen Z, Zhao Y, Ge C, Zhao F, Chen T, Yao M, Li J, Wang H (2011). Genome-wide copy number analyses identified novel cancer genes in hepatocellular carcinoma. Hepatology.

[17] Parashar S, Cheishvili D, Arakelian A, Hussain Z, Tanvir I, Khan HA, Szyf M, Rabbani SA (2015). S-adenosylmethionine blocks osteosarcoma cells proliferation and invasion in vitro and tumor metastasis in vivo: therapeutic and diagnostic clinical applications. Cancer medicine.

[18] Gao JZ, Li J, Du JL, Li XL (2016). Long non-coding RNA HOTAIR is a marker for hepatocellular carcinoma progression and tumor recurrence. Oncol Lett.

[19] Huang S, Qing C, Huang Z, Zhu Y (2016). The long non-coding RNA CCAT2 is up-regulated in ovarian cancer and associated with poor prognosis. Diagn Pathol.

[20] Nor F, Warner K, Zhang Z, Acasigua G, Pearson AT, Kerk S, Helman JI, M Sant'Ana Filho, Wang S, Nor JE (2016). Therapeutic inhibition of the MDM2-p53 interaction prevents recurrence of adenoid cystic carcinomas. Clinical cancer research.

