# Supplementary material for: A novel long noncoding RNA PILRLS promote proliferation through TCL1A by activing MDM2 in Retroperitoneal liposarcoma
Source: Oncotarget. 2017 Jan 25;8(8):13971–8. doi: 10.18632/oncotarget.14814 (PMC5355154; doi:10.18632/oncotarget.14814)
Supplement: Supplementary file 1 [file oncotarget-08-13971-s001.pdf]

A novel long noncoding RNA PILRLS promote proliferation through TCL1A by activing MDM2 in Retroperitoneal liposarcoma

Supplementary Materials

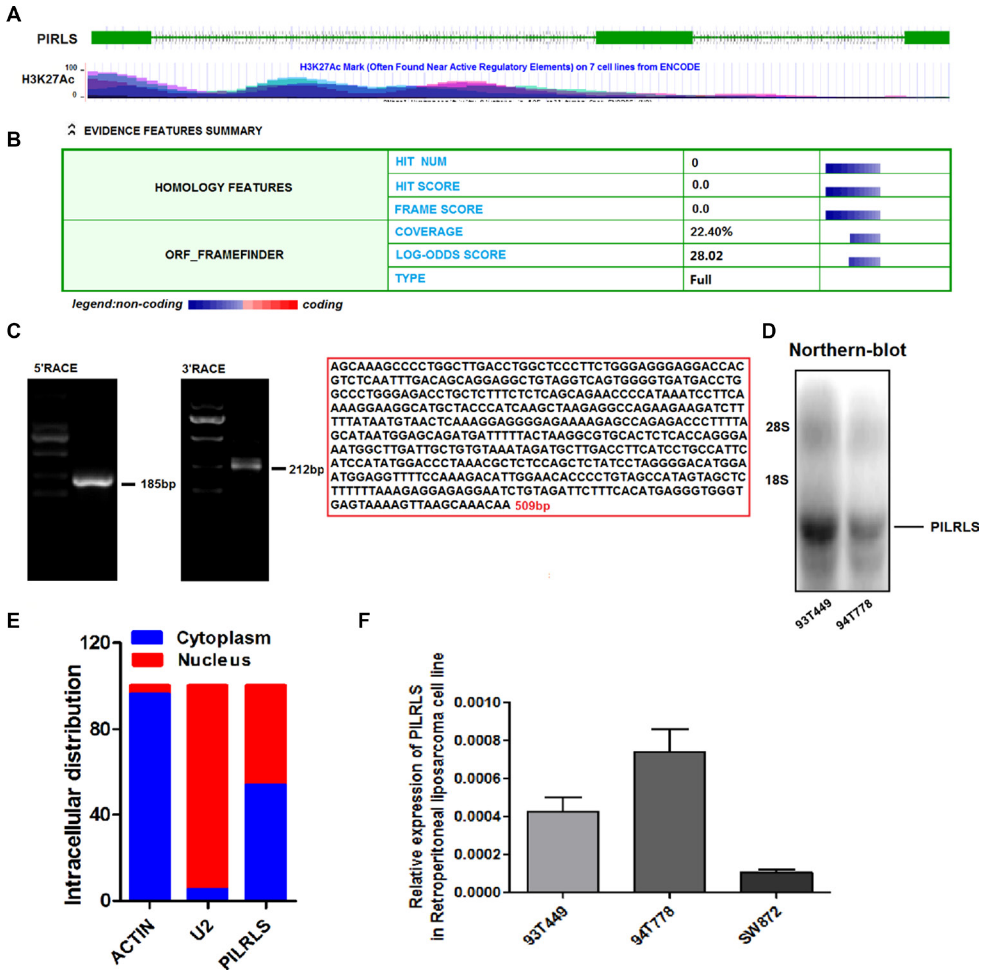

Supplementary Figure 1: Basic characteristic of PILRLS in RLS cells. (**A**) PILRLS located in chr5 with only one transcript. (**B**) Using CPC (Coding Potenital Calculator) software to predicted the protein-coding ability. (**C**) Representative image of PCR products from the 3′ and 5′ RACE and the sequence of PCR products. (**D**) Northern-blot verified the full-length of PILRLS. (**E**) Expression level of PILRLS in cytoplasmic and nuclear extract of RLS cells. U2 as the internal control of nuclear, ACTIN as the internal control of cytoplasmic. (**F**) The expression level of PILRLS in three RLS cell lines.
